# Positron Emission Tomography-Based Response to Target and Immunotherapies in Oncology

**DOI:** 10.3390/medicina56080373

**Published:** 2020-07-24

**Authors:** Maria Isabella Donegani, Giulia Ferrarazzo, Stefano Marra, Alberto Miceli, Stefano Raffa, Matteo Bauckneht, Silvia Morbelli

**Affiliations:** 1 Nuclear Medicine Unit, Department of Health SciencesUniversity of Genoa, 16132 Genoa, Italy; isabella.donegani@gmail.com (M.I.D.); giulia.ferrarazzo@gmail.com (G.F.); marra_ste_pio@yahoo.it (S.M.); albertomiceli23@gmail.com (A.M.); Stefanoraffa@live.com (S.R.); matteo.bauckneht@gmail.com (M.B.); 2 IRCCS Ospedale Policlinico San Martino, 16132 Genoa, Italy

**Keywords:** positron emission tomography, target therapy, immunotherapy, response assessment, PERCIST criteria

## Abstract

2-deoxy-2-[^18^F]fluoro-D-glucose ([^18^F]FDG) is a promising tool to support the evaluation of response to either target therapies or immunotherapy with immune checkpoint inhibitors both in clinical trials and, in selected patients, at the single patient’s level. The present review aims to discuss available evidence related to the use of [^18^F]FDG PET (Positron Emission Tomography) to evaluate the response to target therapies and immune checkpoint inhibitors. Criteria proposed for the standardization of the definition of the PET-based response and complementary value with respect to morphological imaging are commented on. The use of PET-based assessment of the response through metabolic pathways other than glucose metabolism is also relevant in the framework of personalized cancer treatment. A brief discussion of the preliminary evidence for the use of non-FDG PET tracers in the evaluation of the response to new therapies is also provided.

## 1. Introduction

For several decades, cytotoxic chemotherapeutic agents were considered the basis of anticancer treatment for patients with metastatic tumors. Recently, the discovery of molecular origins of tumorigenesis led to the introduction and transition to the clinics of novel agents aiming to target and inhibit signal transduction [1]. By focusing on molecular abnormalities, specific to cancer cells, target cancer therapies have the potential to be more effective against cancer and often less harmful to normal cells than conventional chemotherapeutics [2]. For several reasons, the introduction of these novel types of treatment has been associated to an increased need of predicting patients’ prognosis since baseline evaluation and early capture of the response after initiating therapy. As, especially in patients treated with therapies interfering with signal transduction, these features are strictly related to cancer biology, and functional imaging (Positron Emission Tomography (PET) and magnetic resonance imaging (MRI)) has shown high potential to bring new perspectives in this field especially in terms of response evaluation. In fact, in the frame of target therapies, anatomical response criteria based on the measurement of tumor size by means of computed tomography (CT) might be not able to fully capture viable tumor reduction, thus hampering an early differentiation to responders and non-responders. Moreover, the ongoing revolution in cancer treatment has been unfortunately associated with relevant aggregate costs in terms of cancer care in the last decades [3].

Studies published in the last 10 years have suggested that changes in terms of tumor glucose metabolism as assessed by means of 2-deoxy-2-[^18^F]fluoro-D-glucose ([^18^F]FDG) PET can predict pathologic response rates and patients’ outcomes early in several cancer types and preclinical models [4]. 

In more recent years, the need to tell part responders and non-responders early to limit both toxicity and an unnecessary burden to healthcare systems has become even more prominent after the introduction of compounds targeting intracellular negative regulators, such as immune checkpoint inhibitors (ICPIs) [5]. ICPIs have demonstrated powerful antitumor activity across a wide range of solid tumors [5]. In fact, in parallel with the meaningful effects on patients’ outcome, the hyperactivation of immune systems triggered by immunotherapy has resulted in a wide range of immune-related side effects, triggering inflammatory reactions [6]. Consequently, the radiological effect of immunotherapeutic agents has raised even more relevant and complex challenges for the determination of the imaging-based response at the single patient level [7]. 

PET technology might be a useful tool to support the evaluation of response to either target therapies or ICPIs both in clinical trials and at the single patients’ level; however, several points still need to be addressed to validate the use of PET evaluation in these settings.

The present review aims to summarize and comment on the available evidence related to the use of 2-deoxy-2-[^18^F]fluoro-D-glucose ([^18^F]FDG) PET to evaluate the response to target therapies and ICPIs. Methodological aspects, proposed criteria, as well as its potential added value with respect to morphological imaging are also addressed. Finally, the capability of PET technology to explore several metabolic pathways beyond glucose metabolism is of great potential interest in the field of personalized cancer treatment. Accordingly, a brief discussion of the preliminary evidence for the use of non-FDG PET tracers in the evaluation of response to new therapies is also provided.

## 2. From Tumor Shrinkage to Metabolic Response in Oncoematology: Methodological Overview

The World Health Organization (WHO) criteria were the first radiological criteria introduced into clinical and trial practice to evaluate the dimensional response of solid tumors to cytotoxic therapies [8]. In 2000, the Response Evaluation Criteria in Solid Tumors (RECIST) criteria were also developed, subsequently updated to RECIST v1.1, and are currently widely used [9,10]. WHO and RECIST are standardized and repeatable guidelines based on the quantitative evaluation of tumor size and the number of lesions, and changes on morphological images (CT or MRI). Both systems, by means of bi- and unidimensional measurements, respectively, aim to objectively identify four categories of anatomical responses: Complete response (CR), partial response (PR), stable disease (SD), and progressive disease (PD). The main recognized limits of these methods are related to the challenge of distinguishing and delineating an “active” tumor lesion (viable tissue) from secondary changes (i.e., fibrotic tissue). Moreover, the use of anatomic imaging is suboptimal for tumors that do not change in size early during therapy. Accordingly, these criteria have relevant limits in the evaluation of the response in patients treated with therapies that mainly have a cytostatic effect, such as target therapies [11].

Metabolic imaging with [^18^F]FDG PET and PET/CT has thus become increasingly used to provide biologically relevant information with high prognostic value [12]. The rationale is based on the characteristic strong correlation between [^18^F]FDG PET uptake and the number of viable cancer cells in many tumors, which has been observed to decrease early in responders both to “standard” chemotherapy and target therapies [13]. Although [^18^F]FDG PET is an inherently “quantitative” method potentially providing data on tumor glucose metabolic levels, for a relatively long time, only a binary evaluation based on visual inspection has been used to assess the PET-based tumor response. 

The first proposal for objective PET-based response criteria, the EORTC (European Organization for Research and treatment for Cancer) criteria, was published in 1999. The EORTC metabolic criteria have the historical merit of having conceived the idea of a quantitative (objective) evaluation of the images, going over the visual qualitative one. The EORTC criteria were originally based on the assumption that an early metabolic response can be assessed when anatomical change is not visible yet. The EORTC criteria are based on measurement of the so-called mean standardized uptake value (SUVmean). SUV is defined as the ratio of activity per unit volume of a region of interest (ROI) to the activity per unit whole body volume. SUV aimed to quantify [^18^F]FDG PET uptake in a semiquantitative way as opposed to true quantification through compartmental and kinetic modeling [13]. However, SUV is influenced by multiple factors (not related to the biological characteristics of the tumor or to the patient’s response), such as the uptake time (time between tracer injection and image acquisition), the patient’s glucose levels and body weight, and the dimensions of the lesions and ROIs definition. Accordingly, the EORTC working group has also produced a list of valid recommendations to improve the quality and better standardize [^18^F]FDG PET imaging, thus increasing the standardization of SUV measurement before and after therapy. Nonetheless, for its limits, mostly given to the limited data available at the time, the EORTC criteria have never routinely entered the clinical practice nor were routinely incorporated in clinical trials [12,13].

### 2.1. Metabolic Response in Solid Tumors

In 2009, the PET response criteria in solid tumors (PERCIST) criteria were proposed with the intent of improving the standardization of the PET-based response in oncology. One of the main changes introduced with the PERCIST criteria was the correction of SUV for lean body mass (SUL). This correction aimed to avoid possible effects of SUV measurements due to weight loss occurring during the course of therapy. Moreover, to overcome issues related to the definition and size of ROIs, the PERCIST criteria introduced the use of SULpeak, which is defined after drawing a small standard dimension (a sphere of a 1.2 cm diameter) around the maximal pixel. In fact, SULpeak has the advantage of less statistical variance compared to the single pixel (SUVmax), especially in noisy images. The single hottest tumor lesion then became the target lesion (later on increased to include up to five target lesions before and after therapy).

PERCIST has been demonstrated to be a strong predictor of outcomes and more effective than RECIST in distinguishing responders from non-responders after neoadjuvant chemotherapy in patients treated for esophageal cancer [14,15,16]. Similarly, in advanced non-small-cell lung cancer (NSCLC), after the first cycle of chemotherapy, an [^18^F]FDG uptake reduction was demonstrated to strictly correlate with the time to progression and overall survival (significantly longer for metabolic responders than for non-responders) [17]. In a trial directly comparing PERCIST and RECIST criteria, [^18^F]FDG PET turned out to be more sensitive in detecting CR and progressions in NSCLC patients who received chemotherapy [18,19]. However, PERCIST’s usefulness and advantages over the morphologic criteria still need to be further addressed [14]. Currently, neither EORTC nor PERCIST are used in the daily clinical reporting. Similarly, a supremacy of one over the other has never been demonstrated. Few studies have compared the two PET-based metabolic criteria, generally demonstrating good agreement between the EORTC and PERCIST criteria in evaluating treatment response to solid malignant tumors [20]. The definition of group response based on the EORTC and PERCIST criteria follows the historical classification of the RECIST criteria in terms of a partial and complete response, and stable or progressive disease. These group responses are thus based on variations in terms of tumor metabolism, thus defined as a partial metabolic response, stable metabolic disease, or progressive metabolic disease.

In more recent years, other indicators of cancer-related metabolic activity have been introduced: Metabolic tumor volume (MTV) and total lesion glycolysis (TLG). MTV is a biomarker defined as the volume of tumor tissue that exhibits FDG uptakes above a set SUV threshold [21]. More frequently, two thresholds have been proposed based on iso-counting of the volume of the lesion above 40% of the SUVmax or above a fixed cut-off (generally SUV 2.5) [21]. TLG is the product of MTV and SUVmean, and represents the total activity of all metabolically active cancer cells. These volume-based metrics have the advantage of assessing data from the entire tumor, while the SUVmax or SUVpeak only assess the most active part. On the other hand, both parameters depend on the SUV and therefore are subject to the same limits [22]. Several studies have evaluated the correlation between these parameters and some survival outcomes in different neoplasms. MTV appeared to be superior to SUVmax as a prognostic factor in the overall survival (OS) of patients treated with SBRT in early NSCLC [23]. TLG values were significantly correlated with tumor thickness, depth of invasion, and clinical stage of head and neck cancers and showed a correlation with OS and the presence of distance metastasis [24]. Finally, MTV and TLG demonstrated an early ability to predict OS in patients with colorectal liver metastases post-radioembolization, showing how these parameters can also be used in the assessment of new radiometabolic therapies [22]. 

### 2.2. Metabolic Response in Lymphomas

While use of the PET-based response is promising but not yet incorporated in clinical practice for patients with solid tumors, the metabolic response has been fully validated for both clinical settings and trial use in patients with Hodgkin’s and non-Hodgkin’s lymphomas (HL and NHL).

In fact, the use of CT for assessing response to therapy in HL and NHL suffers from the same limitation described for solid tumors. The CT-based response is hampered by the impossibility to distinguish the presence of fibrosis/necrosis from active neoplastic tissue. In patients with lymphomas, an extensive body of literature has demonstrated the predictive value of the response to treatment both in the early and final evaluation. Given the high predictive value of PET in these specific clinical settings, several criteria have been proposed to standardize response evaluation in patients with HL and NHL both at the interim evaluation and at the end of therapy.

In 2009, the Deauville criteria were fully validated to analyze interim and end-of-treatment PET scans. The Deauville score is based on a visual qualitative scale obtained through the comparison between the lesion’s residual uptake (if any) and the uptake in reference regions (mediastinal blood pool and liver), allowing classification of the residual uptake based on a 5-point scale from 1 (i.e., no uptake) to 5 (i.e., uptake higher than the liver). This criterion was indeed designed to improve the standardization by eliminating the concept of SUV and increasing concordance among readers. [^18^F]FDG PET/CT is currently considered an effective biomarker of lymphomas, and DS criteria became a gold standard included in the Lugano guidelines for the management of the disease as it gives clear clinical information with objectivity and reproducibility [25]. According to the Lugano criteria, the Deauville points match with different types of metabolic response based also on the comparison between baseline and post-therapy scans [26]. [^18^F]FDG PET/CT is now the modality of choice for monitoring and for tailoring response-adapted treatment strategies both for early assessment during therapy (interim PET) and at the end of therapy [27,28]. Group responses based on the RECIST, EORTC, PERCIST, and Lugano criteria are reported in Table 1. 

In conclusion, [^18^F]FDG PET, a widely available tool for imaging both in solid tumor and lymphomas, has showed a promising capability to capture early and atypical patterns of response that might also represent targets to predict response to new therapies.

## 3. PET Response to Target Therapies

The rationale beyond targeted drugs is the inhibition of target proteins that are part of important signal transduction in tumor metabolism, thus interfering with the process of tumor growth, angiogenesis, invasion, and metastasis [4]. Current strategies include antigrowth factor antibodies, receptor antagonists, antireceptor monoclonal antibodies, and small-molecule tyrosine kinase inhibitors. To date, most of these drugs, in addition to having a high economic cost, are only effective in a limited number of patients and are not free from toxicity. 

Therefore, it becomes essential to identify non-responding patients early to improve the cost-effectiveness of these new therapeutic strategies and to limit related toxicities [4].

In this context, the anatomical WHO and RECIST criteria have shown multiple limits. As these criteria were originally validated to reflect tumor shrinkage after chemotherapy, they poorly correlate with other types of cancer treatments characterized by a more prominent cytostatic and/or antiangiogenic effect. This challenge has resulted in the need of adapting these criteria and/or formulating new ones more suitable to capture the response to the variegate effect of target therapies. 

One of the most relevant and historic examples of target therapies whose radiological response was not suitable for a simple shrinkage-based criterion is represented by gastrointestinal stromal tumors (GISTs) [29]. GISTs are the most common mesenchymal tumors of the gastrointestinal tract. GISTs are treated with imatinib mesylate, a biological antineoplastic drug that works by inhibiting a large number of enzymes with tyrosine kinase activity [30]. It is specific for the tyrosine kinase domain c-kit and PDGF-R, commonly mutated in GIST neoplasms. Imatinib was the first biologic drug created to target a specific protein, and after, its use was approved in unresectable and metastatic GISTs [30]. The introduction of this treatment results in a radical improvement of prognosis and therapeutic outcomes of patients [30]. However, imatinib can result in an increase of the lesion size (i.e., due to intratumoral hemorrhage, necrosis, or myxoid degeneration) associated with a marked reduction of tumor metabolism. For these reasons, it soon became clear that the RECIST criteria were inadequate in the response evaluation. In this framework, the input derived from PET studies showing a lack of or shrinkage (or even increase in tumor volume) was paralleled by a marked reduction in tumor metabolism, which has supported the development of new CT-based criteria. In fact, given the inhibitory activity of these biological drugs on several metabolic pathways, the effect of these drugs on glucose metabolism (one of the main metabolic hallmarks of cancer) was somehow expected [4]. It has been shown that the PI3K/AKT/ mTOR pathway directly regulates glucose metabolism and that it is upregulated in many cancers, due to the overexpression of specific oncogenes. mTOR inhibition with a targeted drug leads to a decrease in glucose uptake. This phenomenon justifies the use of metabolic imaging, as a decrease in [^18^F]FDG uptake has been reported on PET scans upon receptor tyrosine kinase and mTOR/PI3K inhibition. The above-mentioned reduction in tumor metabolism is actually associated with a reduction in the density of the lesions (measured as the Hounsfield unit on CT), which is the basis for the criteria developed for the CT-based response in this setting, the so-called Choi criteria. The Choi criteria include not only assessments of the size but also of the density of tumor lesions before and after treatment with imatinib [12]. The response measured with Choi was found to be reproducible, more sensitive, and more precise than RECIST, and was correlated significantly with the time to tumor progression and disease-specific survival [30]. 

[^18^F]FDG PET based on the PERCIST and EORTC criteria have also been previously used in clinical trials in NSCLC patients treated with kinase inhibitors targeting EGFR, such as Erlotinib and Gefitinib [31,32].

Sunaga et al. monitored Gefitinib treatment in a small population of patients using [^18^F]FDG PET PET and reported that an early decrease in lesion tracer uptake is able to predict response. Similarly, Su et al. studied a panel of cell lines with a spectrum of sensitivity to Gefitinib and concluded that [^18^F]FDG PET PET may be a valuable predictor for early response [33]. However, since the tumor can develop secondary mutations of EGFR and other oncogenes, and from the fact that a relevant percentage of metastatic locations differs in genetic expression from the primitive disease, predicting tumor responses solely by the presence of specific mutations is not entirely reliable. 

A recent trial aimed to determine whether early [^18^F]FDG PET was able to predict PFS (Progression Free Survival) and OS in unselected patients with advanced NSCLC using PERCIST criteria on a basal scan, compared with an early scan after 2 weeks of treatment. Again, [^18^F]FDG PET was able to assess changes in tumor [^18^F]FDG uptake predicting PFS and OS in a population of unselected patients, allowing customization of the therapeutic approach in the non-responder and avoiding early discontinuation of the therapy in the responder [34].

Finally, another study prospectively evaluated [^18^F]FDG PET’s role in predicting early response to the neoadjuvant Erlotinib in patients with operable NSCLC. Baseline scans were compared with post-therapy scans acquired as soon as one week of therapy. Patients with a decrease in SUV of 25% or more after one week were classified as responders according to the EORTC criteria. A comparison with the histopathologic examination of the resected specimen was made, showing a good correlation between the metabolic and the pathological response (the latter expressed as the percentage of necrosis observed). This trial’s results supported the concept that early assessment during the course of Erlotinib for NSCLC with [^18^F]FDG PET/CT can identify the response in most patients [33]. 

Finally, a recent study tried to evaluate the performance of [^18^F]FDG PET in predicting HCC tumor progression during Sorafenib treatment, using as an evaluation parameter the SUV ratio between the most hypermetabolic lesion and the liver in the pretreatment scans. [^18^F]FDG PET has been proven to be an independent prognostic factor for survival in patients with HCC receiving Sorafenib, although it may not predict tumor response to the treatment [35]. 

Despite these several lines of evidence, the true value of [^18^F]FDG PET after biological targeted therapies needs to be clarified as many questions remain open [36].

**Figure 1:**
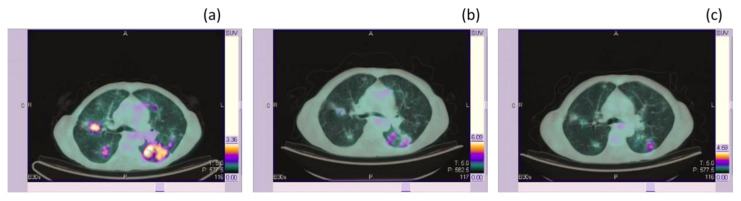
An example of early metabolic response after therapy with Erlotinib in a patient with no smoking history and lung adenocarcinoma. Marked [^18^F]FDG uptake is evident at baseline in bilateral lung nodules (SULpeak 7 in the right inferior pulmonary lobe) (**a**). First response (**b**) three months after therapy initiation highlights a marked reduction of [^18^F]FDG PET uptake in all lung nodules in the absence of a significant reduction in lesion size as evident in the coregistered CT (SULpeak 2 in the right inferior pulmonary lobe, resulting in a partial metabolic response according to the PERCIST criteria while the patient was classified as stable disease according to the RECIST criteria). Nine months after therapy, (**c**) the metabolic response was still clearly evident and was associated with a measurable reduction also in the lesion size (partial response based also on the RECIST criteria). SUL: Standardized uptake value corrected for the lean body mass; [^18^F]FDG: 2-deoxy-2-[^18^F]fluoro-D-glucose; PET: positron emission tomography; CT: computed tomography; RECIST: Response Evaluation Criteria in Solid Tumors.

First, there is no consensus about the time intervals that should be used for the response evaluation and this timeframe might be significantly influenced by the different targets of signal transduction inhibition as well as by tumor biology. The range reported in the literature in trials carrying out [^18^F]FDG PET-based response evaluation after target therapies ranged from 8 days after therapy [30] to several months after therapy, thus suggesting the potential need to validate the best time-point for response evaluation in different clinical settings. Another relevant issue is the lack of standardization regarding which PET-based criteria (i.e., EORTC, PERCIST criteria) should be used to assess response to therapy. In fact, a large variability is reported in studies involving the use of PET in patients treated with target therapies. Several studies have even reported by means of simple visual-qualitative evaluation of [^18^F]FDG PET images without the use of semiquantification. Finally, yet importantly, the real negative predictive value of the [^18^F]FDG PET response in target therapy still needs to be fully defined, and correlation with PFS or OS should be defined for different compounds and tumors [29]. Moreover, given the well-known limits PET in terms of the spatial resolution, a negative PET scan after therapy in patients with solid tumors treated with target therapies might be due to the low amount of viable cells (falling below the PET spatial resolution). Finally, given the effect on the glucose metabolic pathway of some target therapies, at least in some clinical settings, it might be argued if a reduction of FDG PET uptake reflects an effective response to therapy or rather it simply reflects the initial inhibition in the tumor glucose uptake that might not necessary translate into a true clinical response or might be effective only after a longer period of time. This pathophysiological debate is relevant for the definition of a final link between the PET-based response and improved patients’ outcome and survival. Figure 1 shows a representative example of the baseline and post-treatment [^18^F]FDG PET scan in a patient with advanced NSCLC treated with Erlotinib.

## 4. PET to Response to Immunotherapy

### 4.1. Methodological Issues

Immunotherapy has recently emerged as an important advance in cancer treatment. It differs from other strategies, especially from conventional chemotherapy, for a shift in the treatment paradigm. In fact, it promotes an activation of the patient’s immune response rather than being directly cytotoxic on cancer cells [5,37]. In particular, the activation of the immune system to recognize and kill cancer cells is based on different strategies, such as immunomodulatory monoclonal antibodies, directly enhancing the function of components of the immune response against tumor cells, or blocking immunological checkpoints that would otherwise restrain effective antitumor immunity [5]. Most studied molecules used in immunotherapy belong to the category of ICPIs and are directed against the cytotoxic T lymphocyte-associated protein 4 (CTLA-4), against the programmed cell death protein 1 (PD1) or its complex with programmed cell death protein ligand 1 (PD1 /PDL1), which are negative regulators of T cell immune function [38]. In fact, many cancer types show an increased expression of these molecules. Accordingly, compounds for immunotherapy target these molecules, resulting in a downregulation of inhibitory signals, which determines a global augmented activity in the immune system against the tumor cells. In this framework, Ipilimumab, a CTLA-4 inhibitor, was demonstrated to improve survival rates in melanoma patients [39]. Several PD1 /PD-L1 inhibitors have been shown to improve survival rates in patients with different types of tumor, such as lung, melanoma, head and neck, and bladder cancers [40,41,42]. In parallel with these meaningful effects on patients’ outcome, the hyperactivation of immune systems triggered by immunotherapy resulting in a wide range of side effects has been reported, including rash, myalgia, arthritis, enterocolitis, thyroiditis, hypophysitis, and pancreatitis [6]. Similar mechanisms underlie a variegate effect of neoplastic lesions’ size and number that might hamper the CT-based evaluation of response to immunotherapy in a subgroup of patients [37]. 

In particular, one potential challenge in the evaluation of response to ICPIs is represented by the so-called pseudoprogression. This phenomenon consists on an initial increase in the tumor volume and/or number of lesions (due to inflammatory cells’ infiltration that mimics cancer progression) followed by the demonstration of tumor shrinkage and a subsequent positive effect in terms of patients’ outcome [43]. Pseudoprogression is indeed determined by the activation of the immune system that starts to surround the tumor [44]. Pseudoprogression has been reported, especially after therapy with CTLA-4 inhibitors in patients with melanoma [7], and less frequently in other tumor types (possibly in less than 5% of patients with other diseases, including NSCLC and lymphoma) [43]. Accordingly, if the response is based on the conventional RECIST criteria, these patients may initially meet the conventional response criteria for PD but later might show a reduction in the tumor burden and a final favorable outcome. Accordingly, conventional-based CT has been modified to overcome this limitation by the creation of an immune-related response (irRC) and immune-RECIST criteria [45,46]. Actually, in the presence of response-related inflammatory cells’ infiltration, patients’ assessment based on [^18^F]FDG PET might also result in findings suggestive of a pseudo (or lack of) response. In fact, one of the most commonly encountered false-positive PET/CT interpretation pitfalls is related to an increased FDG uptake due to inflammation, especially after recent chemo-radiotherapy and/or surgery. Despite the fact that inflammatory infiltration and related tumor changes can also hamper the reliability of the [^18^F]FDG PET-based response, several studies have to date suggested a potential added value of [^18^F]FDG PET in a subset of patients [43]. 

### 4.2. PET-Based Response to ICPIs in Patients with Melanoma

Preliminary experience of the metabolic response to immunotherapy in metastatic melanoma patients was published in 2015 by Sachpekidis and colleagues [47]. This study evaluated the capability of the EORTC response classification to predict patients’ outcome after two cycles of treatment with ipilimumab in 22 patients with melanoma. Early PET evaluation was demonstrated to be predictive of late response and of the overall outcome both in patients with stable metabolic disease and patients with progressive metabolic disease. 

Moreover, in another study carried out by Kong and colleagues in patients with metastatic melanoma, a measurable metabolic response was able to predict a prolonged response to anti-PD1 treatment [48]. In this study among the 12 patients showing a negative PET scan, 6 were characterized by residual disease at the CT scan and 5 even stopped the treatment but none of them showed recurrence in the 6-10 months of further follow-up. Similarly, Cho and colleagues [46] recruited 20 patients with metastatic melanoma in therapy with either ipilimumab or nivolumab and performed both an early assessment (after 18-21 days) and late assessment (after 4 months) by means of the RECIST, PERCIST, and EORTC criteria in order to define the best matching criteria for PET scan evaluation. While in the early assessment, a low inter-criteria agreement has been reported, late scans resulted in an excellent agreement. In 2017, Amrane et al. aimed to define the best match between morphological and metabolic responses in 20 patients with advanced melanoma treated with ipilimumab (*n* = 1 7) or nivolumab (*n* = 3) and developed the so-called PECRIT criteria (PET/TC Criteria for Early Prediction of Response to Immune Checkpoint Inhibitor Therapy). Of note this classification introduces the clinical benefit into the definition of response, thus further suggesting that the presence of pseudoprogression might be suspected when radiological progression is paralleled by an evident improvement in the clinical performance status [49]. Immunotherapy-modified PERCIST (imPERCIST) criteria have also been proposed but not yet fully validated in patients with melanoma treated with ipilimumab [50]. According to the imPERCIST criteria, the appearance of new lesions alone did not result in progressive metabolic disease (PMD) and thus, PMD is defined only by an increase of the sum of SULpeaks by 30%. Similarly, new lesions are included in the sum of the SULpeak if they show higher uptake than existing target lesions or if fewer than five target lesions are detected on the baseline scan. Preliminary experience on the imPERCIST criteria is also available in patients with NSCLC treated with Nivolumab. 

### 4.3. PET-Based Response to ICPIs in Patients with NSCLC

Rossi and colleagues aimed to compare the evaluation of the first response to Nivolumab by means of CT-based and PET (PERCIST and imPERCIST) criteria in 48 patients with advanced NSCLC [51]. Low concordance was highlighted between the CT- and PET-based criteria (both PERCIST and imPERCIST versus RECIST and irRC, respectively). However, IrRC was more reliable in distinguishing responders from non-responders, but, thanks to the prognostic value of the partial metabolic response, the PET-based response maintained prognostic significant in patients classified as progressive disease on the basis of irRC. Taken altogether, these results do not support the routine use of [^18^F]FDG PET in the general population of NSCLC patients treated with ICIPs, but they suggest the added prognostic value of the metabolic response assessment, potentially improving therapeutic decision-making. 

**Figure 2:**
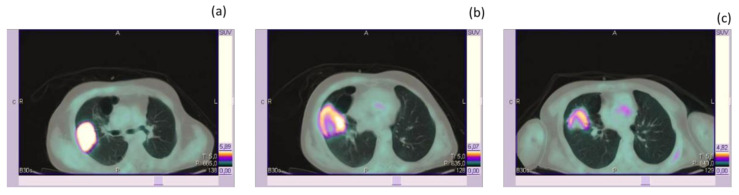
A representative example of metabolic response in patients with advanced NSCLC treated with Nivolumab. Baseline [^18^F]FDG PET/CT (**a**) shows a right parascissural lung lesion (dd max 7 cm; SUVmax 12). Only a mild reduction in lesion size was highlighted at the first response evaluation (2 months after therapy; **b**); however, a more marked metabolic reduction was already evident (SUVmax 6). Of note, a metabolic active volume reduction was even more evident than a SUVmax reduction as a central photopenic area was evident after treatment. The first metabolic response was able to predict the patient’s response evolution, as after a further two months, both lesion size and metabolism were further decreased (SUVmax 4, **c**). NSCLC: Non Small Cell Lung Cancer.

Interestingly enough, studies have already been performed in order to determine which metabolic criteria could better define the response of HL to immunotherapy; however, no conclusive data are available to date to define which criteria should be used to assess the PET metabolic response in this setting [52]. Finally, given the capability of [^18^F]FDG PET/CT to highlight the presence of hypermetabolism related to inflammation, it has also been used to capture the presence of immune-related adverse events (IRAEs) and to correlate them with patients’ outcome [53]. PET-detectable IRAE was useful to predict a favorable outcome. In a retrospective study, patients with malignant melanoma, malignant lymphoma, and renal cell carcinoma treated with immunotherapy were evaluated. Patients with IRAE showed a better outcome (with 9 out of 11 patients with IRAE showing a complete response at the final evaluation). On the other side, the potential confounding effect of hypermetabolic lesions due to IRAE should be taken into account when reporting [^18^F]FDG PET in patients treated with immunotherapy. Sarcoid-like lung lesions and reactive lymph nodes have been reported and should not be confounded with PMD [53,54,55]. Similarly, inversion of the liver-to-spleen ratio (normally >1), reflecting immune activation preceding T cell proliferation, has been reported [55]. In conclusion, despite the more effective way of assessing the [^18^F]FDG PET-based response in patients treated with ICPIs, available studies suggest that [^18^F]FDG PET can support decision-making about the continuation/discontinuation of therapy, as it can open several windows able to capture different aspects associated with the effect of treatment (i.e., pseudoprogression, hyperprogression, and IRAE) [56]. Table 2 reports the characteristics of published studies involving the use of [^18^F]FDG PET to assess the response to ICI [46,47,48,50,51,54,57,58,59,60,61,62,63,64,65,66,67,68]. Figure 2 shows a representative example of baseline and post-treatment [^18^F]FDG PET in a patient with advanced NSCLC treated with Nivolumab.

## 5. Response Assessment and Prediction with NON-FDG Tracers

### 5.1. [11. C]choline, [^18^F]choline, and [^68^Ga]PSMA

As mentioned, although not yet validated, several ongoing efforts are trying to explore (and possibly standardize) the use of objective criteria to report [^18^F]FDG PET/CT in patients treated with target therapies. By contrast, very few studies addressed the capability of standardized PET response evaluation to target (and either conventional) therapies carried out with non-FDG PET tracers. A preliminary experience is available for [^11^C]choline PET/CT. De Giorgi et al. [69,70] assessed the usefulness of [^18^F]choline PET/CT for evaluating early response to abiraterone and enzalutamide in mCRPC patients. A radiologic response was associated with more favorable overall survival than a PSA response of greater than or equal to 50% alone. Maines et al. [71] evaluated [^18^F]choline PET/CT in monitoring response to enzalutamide in 30 mCRPC patients. SUVmax measured at baseline before enzalutamide was significantly related to radiologic progression-free survival and overall survival. Middendorp et al. [72] reported that response evaluation based on [^18^F]choline PET/CT results after tyrosine kinase inhibitor treatment was effective to predict outcome in two renal cell carcinoma patients. Similarly, Kitajima et al. [73] performed 34 scans before/after a combined total of 17 courses of treatment, including molecular target therapy and immunotherapy, in 6 patients with prostate cancer and 2 with renal cell carcinoma. [^11^C]choline PET/CT was useful for detecting viable residual tumors and evaluating the treatment response, showing a better treatment response than CT. In more recent years, PSMA, a transmembrane glycoprotein highly expressed on the cell surface of prostate cancer cells, with expression increasing with more aggressive cancer types and in castration-resistant disease, has emerged as a compound of utmost importance for imaging and therapy of prostate cancer [74]. In the last years, PET with [^68^Ga]PSMA has become increasingly proposed especially for the early and accurate detection of disease relapse [74]. Its role in post-treatment settings is less defined; however, the possibility of labelling PSMA with the beta-emitter ^177^Lu has opened a further therapeutic opportunity in prostate cancer patients whose effect can be evaluated by means of [^68^Ga]PSMA PET [75,76,77]. Indeed, PSMA PET has a role in predicting treatment response to [^177^Lu]PSMA therapy and in identifying subsequent patterns of failure, determining the next best treatment options [75,76,77]. A minimal PSMA receptor density threshold is required to get any treatment response while factors that determine treatment response to [^177^Lu]PSMA therapy are homogeneity of PSMA receptor expression across cancer cells, radiation sensitivity, and a hypoxic status of these cells [78]. Identifying patterns of response or failure on PSMA imaging after [^177^Lu]PSMA therapy may, in the near future, to determine subsequent treatment options after or in addition to [^177^Lu]PSMA therapy [79].

### 5.2. [68. Ga]DOTA-Conjugate Peptides

Even before the availability of target and radiometabolic options for patients with advanced prostate cancer, another pathway has been significantly investigated and used in the field of nuclear medicine both for diagnostic and treatment purposes. The somatostatin receptor (SSTR), expressed by most neuroendocrine neoplasms (NENs), can in fact be the targets for radionuclide imaging and therapy. [^68^Ga]DOTA-conjugate peptides (DOTA-TATE, DOTA-TOC, and DOTA-NOC) PET/CT are used to determine SSTR status (patients with SSTR-positive tumors are more likely to respond to targeted somatostatin analogue therapy), to predict therapeutic response to peptide receptor radionuclide therapy (PRRT) and to monitor the response to PRRT [80]. PRRT is successfully used to target metastatic or inoperable neuroendocrine tumors expressing subtype 2, leading to a longer survival and improved quality of life [81,82]. Evaluation of the response to treatment includes consideration of the morphological and PET/SPECT functional status. Functional imaging is a valuable instrument to assess the course of the disease, being able to predict morphological response. Combined functional and morphological imaging may in many cases better reflect the true behavior of the tumor following PRRT. However, functional imaging is not yet accepted as a substitute for morphological imaging to assess treatment tumor response [81]. 

### 5.3. Other Non-FDG Tracers

Finally, the ductility of PET technology has allowed the development of radiolabeled compounds able to directly track targets of target therapies in specific clinical settings. 16α-[^18^F]fluoro-17β-estradiol ([^18^F]FES) and 16β-[^18^F]fluoro-5α-dihydrotestosterone ([^18^F]FDHT) PET/CT allow visualization, respectively, of estrogen (ER) and androgen receptor (AR) status in tumor lesions. [^18^F]FES and [^18^F]FDHT uptake correlates well with ER and AR expression levels in breast and prostatic cancer [83,84,85]. Multiple tumor sites throughout the body can be evaluated without patient discomfort, even sites that would be challenging to reach by needle biopsy and sites where tissue processing can affect the ER assay. Thus, intertumoral heterogeneity can be detected by [^18^F]FES-PET [86,87,88], being predictive of a benefit from endocrine therapy [89]. [^18^F]FES PET can be used to assess residual ER availability during treatment [90]. In patients with prostate cancer, [^18^F]FDHT PET was used to determine the optimal dose of the AR blocker enzalutamide in a phase 1 trial [91]. FES also represents the standard in vivo probe for pharmacodynamic target occupancy studies used to determine appropriate dosing of currently used ER antagonists [90,92].

In recent years, new and extremely promising non-FDG PET tracers have also been evaluated in the field of immunotherapy, with the aim of predicting response to ICPIs even before treatment [93,94]. In fact, PD-L1 expression by immunohistochemistry has been correlated with response and survival following PD-(L)1 monoclonal antibody therapy in patients with NSCLC [92]. However, a lack of response has also been demonstrated in patients with PD-L1 expression and has been linked to heterogeneity of PD-L1 expression within tumors. PET studies in preclinical models have tested this hypothesis. Indeed Niemeijer et al. and Bensch et al. reported the first-in-human results of whole body PET imaging by means of ^18^F-BMS-986192, ^89^Zr-Nivolumab, and ^89^Zr atezolizumab prior to treatment with ICPIs in patients with different tumor types and, highlighted with whole body PD-(L)1 PET-CT, a significant tumor tracer uptake heterogeneity both in different patients and in different tumor lesions in the same patient [93,94]. 

## 6. Conclusions

In conclusion, new and emerging therapeutic strategies in clinical oncology with a prominent cytostatic rather than cytotoxic effect have suggested a possible inadequacy of established morphologic size-based criteria for the assessment of tumor response in a subset of patients. Functional and molecular parameters for imaging are under intense investigation. In this framework, [^18^F]FDG PET, a widely available tool for imaging in oncology, has showed promising features in capturing an unexpected pattern of response as well as for the identification of non-responders. The added value of [^18^F]FDG PET can thus be of interest not only to avoid unnecessary toxicity (in non-responders) but also to support a treatment flow-chart that is sustainable for the healthcare system. In this regard, there is an urgent need to further validate and practically implement specific criteria for an objective evaluation of the PET-based response to new therapies. As a more systematic approach, [^18^F]FDG PET might also pave the path for a structured use of non-FDG PET tracers, thus completing exploiting the potential of PET technology in the emerging field of personalized cancer medicine.

## Figures and Tables

**Table 1:** A schematic summary of the class of response according to RECIST 1.1, EORTC, PERCIST, and Lugano criteria.

Category	RECIST 1.1	EORTC (1999)	PERCIST	LUGANO
**Target lesion**	Up 2 per organs, maximum 5 in total	The most [^18^F]FDG avid lesions (SUV BSA). Number of lesions not specified	The hottest single tumor lesion at baseline [^18^F]FDG PET (SUL peak)	Up to 6 measurable nodal (LDi >1.5 cm) and extranodal sites (LDi >1cm) Non-measurable disease sites LDi >1.5 cm LDi >1.0 cm All other disease sites nodal/extranodal/assessable disease (skin, GI, bone, spleen, liver, kidneys, effusions)
**New lesion**	Any new lesion results in progressive disease at first appearance			
**Complete response**	Disappearance of all target and non-target lesions Nodes must regress to < 10 mm short axis No new lesions Confirmation is required	Complete absence of [^18^F]FDG uptake	Complete resolution of [^18^F]FDG uptake within the target lesion (< mean liver activity and indistinguishable from background/blood pool and no new [^18^F]FDG avid lesions)	DS 1, 2, and 3 in nodal or extra-nodal sites, with or without residual mass
**Partial response**	≥30% decrease in tumor burden compared to baseline Confirmation required	A decrease in SUV > 25%	A reduction of a minimum of 30% in the target tumor [^18^F]FDG SUL peak PMR	DS 4 or 5 with [^18^F]FDG uptake decreased compared with baseline
**Progressive disease**	≥20% + 5 mm absolute increase in tumor burden compared with nadir Appearance of new lesions or progression of non-target lesions	An increase in SUV > 25% or appearance of new lesions	A 30% increase in [^18^F]FDG SUL peak or advent of new [^18^F]FDG avid lesions	DS 4 or 5 with an increase in uptake from baseline &/or new lesions
**Stable disease**	Neither partial response nor progressive disease	Increase in SUV by < 25% or decrease in SUV by < 15%	Disease other than CMR, PMR or PMD	DS 4 or 5 with no change in [^18^F]FDG uptake

RECIST: Response Evaluation Criteria in Solid Tumors; PERCIST: PET response criteria in solid tumors; EORTC: European Organisation of Research and Treatment for Cancers; [^18^F]FDG: 2-deoxy-2-[^18^F]fluoro-D-glucose; SUV: standardized uptake value; SUL: Standardized uptake value corrected for the lean body mass; PMD: progressive metabolic diseas; PMR: partial metabolic response; CMR: complete metabolic response; BSA: body surface area; DS: Deauville Score; LDI: lawer diameter; GI: gastrointestinal tract.

**Table 2:** Studies evaluating the role of FDG PET in patients treated with immune checkpoint inhibitors.

Reference	Study Type	Patients’ Characteristics	Aims	Methods	Results	Conclusions
Sachpekidis et al. [47]	prospective	22 patients suffering from unresectable metastatic melanoma, scheduled for ipilimumab treatment.	To evaluate the role of [^18^F]FDG PET/CT performed after two cycles of ipilimumab in predicting the final response to therapy.	PET/CT scanning was performed before the start of treatment (baseline scan), after two cycles of treatment (early response) and at the end of treatment after four cycles (late response). Evaluation of the patient response to treatment on PET was based on EORTC criteria.	Early PET/CT performed after two ipilimumab cycles predicted treatment response in 13 of the 15 PMD patients, in five of the five SMD patients and in neither of the two PMR patients.	[^18^F]FDG PET/CT after two cycles of ipilimumab is highly predictive of the final treatment outcome in patients with PMD and SMD.
Kong et al. [48]	prospective	27 patients with unresectable stage IIIC or IV melanoma after prolonged treatment with anti-PD-1 antibodies.	To examine the hypothesis that patients with prolonged response to treatment may have metabolically inactive lesions by [^18^F]FDG PET/CT.	Scans were performed at a median of 15.2 months (range 12-29 months) after starting treatment.	8 patients with positive scans underwent biopsy; 5 of 8 (62%) were melanoma and 3 of 8 (38%) were immune cell infiltrates. Of the 12 patients with negative [^18^F]FDG PET scans, 6 had residual computerized tomography-visible lesions, 5 have ceased treatment, and none have recurred with follow-up of 6-10 months.	Patients with residual metastases after a prolonged period without progression on anti-PD-1 therapy may have metabolically inactive lesions. Isolated metabolically active lesions in clinically well patients may reveal immune cell infiltrates rather than melanoma.
Cho et al. [46]	prospective	20 patients with advanced melanoma receiving ICIPs.	To evaluate [^18^F]FDG PET/CT scanning as an early predictor of response to immune checkpoint inhibitors in patients with advanced melanoma.	[^18^F]FDG PET/CT was performed at 3 scan intervals. Tumor response at each posttreatment time point was assessed according to RECIST 1.1, PERCIST 1.0 and EORTC criteria.	Early response evaluations using RECIST 1.1, immune-related response criteria, PERCIST, and EORTC criteria demonstrated accuracies of 75%, 70%, 70%, and 65%, respectively. By combining early anatomic and functional imaging data criteria to predict eventual response were developed.	Combining functional and anatomic imaging parameters from [^18^F]FDG PET/CT scans performed early during immunotherapy appears predictive for eventual response in patients with advanced melanoma.
Dercle et al. [49]	retrospective	16 heavily pre-treated patients with Hodgkin’s Lymphoma (HL).	To define the depth and time of maximal anti-tumor response to anti-PD1 in heavily pre-treated patients with HL.	The [^18^F]FDG PET/CT and CT data of all relapsed or refractory HL were reviewed according to the International Harmonisation Project Cheson 2014 criteria and the LYRIC criteria.	Fifty-six percent of patients (9/16) achieved an objective response at 3 months, including 19% (3/16) of complete response. Seventeen percent (1/6) of partial responders at 3 months were converted in a complete response. 22% (2/9) of responders at 3 months relapsed before one year. The nadir was reached at 12.7 (3.0-23.0) months. The median (range) depth of response at nadir was -77% (-50% to 100%).	Complete metabolic responses occurred within 6 months, a minority of partial responses were converted in complete response, and the median nadir was observed one year after treatment initiation. These data could help to better define the optimal treatment strategy by PET or CECT-directed approaches
Tan et al. [57]	retrospective	140 metastatic melanoma patients treated with anti-PD-1-based immunotherapy with baseline and 1-year [^18^F]FDG PET and CT imaging.	To investigate whether [^18^F]FDG PET may better predict long-term outcomes compared with CT	One-year response was determined using RECIST for CT and EORTC criteria for PET. PFS was determined from the 1-year landmark.	Whilst only a small proportion of patients have a CR at 1 year, most patients with a PR have CMR on PET. Almost all patients with CMR at 1 year have ongoing response to therapy thereafter.	PET may have utility in predicting long-term benefit and help guide discontinuation of therapy.
Ito et al. [50]	retrospective	60 patients with metastatic melanoma who underwent [^18^F]FDG PET/CT scans both before and after ipilimumab therapy.	To evaluate the association between tumor response on [^18^F]FDG PET/CT and prognosis in patients with metastatic malignant melanoma treated with ipilimumab.	Tumor response was assessed by the change in the sum of SULpeak of up to 5 lesions according to PERCIST5. New lesions on PET that appeared suggestive of metastases were considered PMD. An immunotherapy-modified response classification was also evaluated (imPERCIST5).	In responders and non-responders, the 2-y OS was 66% versus 29% for imPERCIST5 (*p* = 0.003). After multivariate analysis, imPERCIST5 remained prognostic (*p* = 0.005). New sites of focal [^18^F]FDG uptake occurred more often in patients with PMD (*n* = 24) by imPERCIST5 than in those with stable metabolic disease (*n* = 7) or partial metabolic response (*n* = 4).	In patients with metastatic melanoma treated with ipilimumab, tumor response according to PERCIST was associated with OS. Our data suggest that PMD should not be defined by the appearance of new lesions, but rather by an increase in the sum of SULpeak.
Jreige et al. [58]]	retrospective	49 patients with confirmed NSCLC.	To investigate correlation between [^18^F]FDG PET/CT-based markers and tumor tissue expression of PD-L1, necrosis and clinical outcome in patients treated with ICPIs	SUVmax, SUVmean, MTV and TLG were obtained from [^18^F]FDG PET/CT images. Metabolic-to-morphological volume ratio (MMVR) was measured.	All tumors showed metabolic [^18^F]FDG PET uptake. MMVR was correlated inversely with PD-L1 expression in tumor cells. Furthermore, PD-L1 expression and low MMVR were significantly correlated with clinical benefit. Necrosis was correlated negatively with MMVR.	MMVR was introduced as a new imaging biomarker and its ability to noninvasively capture increased PD-L1 tumor expression and predict clinical benefit from checkpoint blockade in NSCLC should be further evaluated.
Amrane et al. [59]	retrospective	37 patients with unresectable metastatic cutaneous melanoma eligible for immunotherapy.	To assess serial [^18^F]FDG PET/CT imaging according to morphological and functional to predict clinical response to therapy in patients with advanced melanoma receiving immune checkpoint blocking agents.	Among 37 assessed patients, 27 had 1 line of ICI, 8 had 2 lines of ICI and 2 patients had 3 lines of ICI: total of 49 PET/CTs.	Median PFS was 29.62 months (*p* = 0.001: RECIST 1.1), (*p* < 0.0001: iRECIST), (*p* = 0.000: PERCIST), (*p* = 0.072: PECRIT). Median OS was 36.62 months (*p* = 0.005: RECIST 1.1), (*p* < 0.0001: iRECIST), (*p* = 0.001: PERCIST), (*p* = 0.082 PECRIT).	[^18^F]FDG PET/CT scans could detect eventual ICI-response in patients with metastatic melanoma. According to our study, iRECIST and PERCIST 1.0 may provide the most optimal ICI-related response classification.
Rossi et al. [51]	prospective	72 patients with advanced NSCLC.	To compare the evaluation of first response to Nivolumab by means of CT-based criteria with respect to [^18^F]FDG PET response criteria in NSCLC patients.	Patients underwent CT scan and FDG-PET at baseline and after 4 cycles (first evaluation). Response was evaluated with CT scan by means RECIST 1.1 and IrRC and with FDG-PET by means of PERCIST and imPERCIST criteria. The concordance between CT- and PET-based criteria and the capability of each method to OS were evaluated.	A low concordance between CT- and PET-based criteria was observed. Looking at OS, IrRC were more reliable to distinguish responders from non-responders. However, thanks to the prognostic value of partial metabolic response assessed by both PERCIST and Immuno-PERCIST, PET-based response maintained prognostic significant in patients classified as progressive disease on the basis of IrRC.	The added prognostic value of the metabolic response assessment, potentially improving the therapeutic decision-making was suggested.
Castello et al. [60]	prospective	50 NSCLC patients treated with ICIs.	To investigate the prevalence of such a phenomenon and to assess its association with clinical variables and metabolic parameters by [^18^F]FDG PET/CT.	All patients underwent contrast-enhanced CT, [^18^F]FDG PET/CT, and complete peripheral blood sampling at baseline before ICI treatment. A Cox proportional hazards regression analysis was used to evaluate factors independently associated with OS.	Survival analysis showed a median OS of 4 months for the HPD group, compared with 15 mo for the non-HPD group (*p* = 0.003). Median OS was significantly different when all the response categories were considered. Multivariate analysis identified MTV and derived neutrophil-to-lymphocyte ratio as independent predictors for OS.	The use of ICIs might represent a concern in patients with high metabolic tumor burden and inflammatory indices at baseline.
Annovazzi et al. [61]	retrospective	57 patients with metastatic melanoma treated with ipilimumab or with PD-1 inhibitors who performed an [^18^F]FDG PET/CT scan before treatment and 12 to 18 weeks later.	To compare the diagnostic accuracy of different [^18^F]FDG PET/CT criteria to predict therapy response and clinical outcome in melanoma patients treated with immune checkpoint inhibitors.	Response at PET1 was evaluated according to RECIST 1.1, EORTC, PERCIMT, and by percentage change of (MTV) and TLG of up to 5 target lesions. Performance of each criterion at PET1 to predict clinical benefit at 6 months since starting immunotherapy was assessed and correlated to PFS.	The best predictor of therapy response was MTV combined with PERCIMT criteria (accuracy, 0.96). In group 2, overlapping results were found for EORTC, MTV, and total lesion glycolysis (accuracy, 0.97). The reliability of the above parameters was also confirmed in the progression-free survival analysis.	[^18^F]FDG PET/CT performed after 3 to 4 months since starting immunotherapy can correctly evaluate response to treatment and can also able to predict long-term clinical outcome. Performance of [^18^F]FDG PET/CT and criteria for response assessment is influenced by the class of treatment.
Castello et al. [62]	prospective	35 NSCLC patients	To examine CTC count and its association with metabolic parameters and clinical outcomes in NSCLC patients treated with ICI.	All patients underwent an [^18^F]FDG PET/CT scan and CTC detection through Isolation by Size of Tumor/Trophoblastic Cells (ISET) from peripheral blood samples obtained at baseline and 8 weeks after ICI initiation. Association of CTC count with clinical and metabolic characteristics was studied.	ΔCTC was significantly associated with tumor metabolic response set by EORTC criteria (*p* = 0.033). At the first restaging, patients with a high tumor burden, that is, metabolic tumor volume (MTV) and total lesion glycolysis (TLG), had a higher CTC count (*p* = 0.009). Multivariate analysis identified CTC count at 8 weeks as an independent predictor for PFS and OS, whereas ΔMTV and maximum standardized uptake value variation (ΔSUVmax) was predictive for PFS and OS, respectively.	CTC number is modulated by previous treatments and correlates with metabolic response during ICI. Moreover, elevated CTC count, along with metabolic parameters, are prognostic factors for PFS and OS.
Hashimoto et al. [63]	retrospective	85 patients with previously treated NSCLC who underwent [^18^F]FDG PET just before administration of nivolumab or pembrolizumab.	To retrospectively examine the prognostic significance of [^18^F]FDG uptake as a predictive marker of anti-PD-1 antibody.	MTV, TLG and SUVmax on [^18^F]FDG uptake were assessed.	The tumor metabolic activity by TLG and MTV was identified as an independent prognostic factor for predicting outcome after anti-PD-1 antibody therapy.	TLG and MTV on [^18^F]FDG uptake may predict the prognosis after anti-PD-1 antibodies in patients with previously treated NSCLC.
Seban et al. [64]	retrospective	56 patients with non-resectable mucosal melanoma (Muc-M) or cutaneous melanoma (Cut-M) who underwent baseline [^18^F]FDG PET/CT before treatment with ICIs.	To compare the prognostic value of imaging biomarkers derived from a quantitative analysis of baseline [^18^F]FDG PET/CT in patients with mucosal melanoma (Muc-M) or cutaneous melanoma (Cut-M) treated with ICIs.	Parameters were extracted from (i) tumoral tissues: SUVmax, SUVmean, TMTV and TLG and (ii) lymphoid tissues: BLR and SLR. Association with survival and response was evaluated using Cox prediction models,	In Muc-M, increased tumor SUVmax was associated with shorter OS while it was not correlated with PFS, ORR, or DCR. In Cut-M, increased TMTV and increased BLR were independently associated with shorter OS, shorter PFS, and lower response (ORR, DCR).	For Muc-M patients treated with ICI, the only prognostic imaging biomarker was a high baseline maximal glycolytic activity (SUVmax), whereas for Cut-M patients, baseline metabolic tumor burden or bone marrow metabolism was negatively correlated to ICI response duration.
Nakamoto et al. [65]	retrospective	85 melanoma patients treated with ICIs who underwent PET/CT scans before and approximately 3 months after the start of immunotherapy.	To investigate the prognostic value of MTV and other metabolic tumor parameters, obtained from baseline and first restaging [^18^F]FDG PET/CT scans in melanoma patients treated with ICIs.	Metabolic tumor parameters including MTV for all melanoma lesions were measured on each scan. A Cox proportional hazards model was used for univariate and multivariate analyses of metabolic parameters combined with known clinical prognostic factors associated OS.	MTV obtained from first restaging PET/CT scans (MTVpost) was the strongest prognostic factor for OS among PET/CT parameters (*p* < 0.0001). The median OS in patients with high MTVpost (≥ 23.44) was 16 months as compared with more than 60 months in patients with low MTVpost (*p* = 0.0003).	Whole-body metabolic tumor volume from PET scan acquired approximately 3 months following initiation of immunotherapy (MTVpost) is a strong prognostic indicator of OS in melanoma patients.
Iravani et al. [66]	retrospective	31 patients who had first-line nivolumab plus ipilimumab; pre- and post-treatment [^18^F]FDG PET/CT scans within 2 and 4 months of starting ICI, respectively and at least one lesion assessable by PERCIST.	To investigate the role of [^18^F]FDG PET/CT in monitoring of response and immune-related adverse events following first-line combination-ICI therapy for advanced melanoma.	Outcomes in patients who had first-line nivolumab plus ipilimumab were reviewed; pre- and post-treatment FDG-PET/CT scans within 2 and 4 months of starting ICI, respectively; and at least one lesion assessable by PERCIST.	The best-overall responses were CMR in 25 (80%), PMR in 3 (10%), and PMD in 3 (10%) patients. Patients with PMD had significantly higher pre-treatment wbMTV (*p* = 0.009). Secondary progression The most common [^18^F]FDG PET/CT detectable immune-related adverse event were endocrinopathies and enterocolitis.	[^18^F]FDG PET/CT response evaluation predicts the long-term outcome of patients treated with first-line combination-ICIs.. Beyond response assessment, [^18^F]FDG PET/CT frequently detects clinically relevant irAEs.
Umeda et al. [67]	prospective	25 with previously treated NSCLC	To determine whether changes in integrated [^18^F]FDG PET/MRI parameters after the first 2 weeks of antiprogrammed death-1 antibody nivolumab therapy could predict the response of patients with NSCLC.	Patients underwent [^18^F]FDG PET/MRI before and at 2 weeks after nivolumab therapy. Changes in SUVmax, ΔTLG and ΔADC between the two scans were calculated and evaluated for their associations with the clinical response to therapy.	Non-PD patients had significantly decreased TLG, increased ADCmean and lower ΔTLG + ΔADCmean than PD patients.	A combination of ΔTLG and ΔADCmean measured by integrated [^18^F]FDG PET/MRI may have value as a predictor of the response and survival of patients with NSCLC following nivolumab therapy.
Castello et al. [68]	prospective	20 NSCLC patients candidate to ICI therapy.	To investigate the role of sPD-L1 in NSCLC patients treated with ICI and to analyze its association with clinical outcomes and metabolic parameters by [^18^F]FDG PE T/CT.	Patients who had serum frozen samples and [^18^F]FDG PET/CT available, both at baseline and at the first restaging after approximately three or four cycles of ICI, were included. Before and after 3–4 cycles of ICI, peripheral blood samples were collected from patients.	A significant association between patients with elevated sPD-L1, above the median value, and high metabolic tumor burden, expressed by MTV (*p* = 0.034) and TLG (*p* = 0.049) was found. At the first restaging after 7–8 weeks, median sPD-L1 levels significantly increased as compared to baseline median value (*p* = 0.017).	The association between metabolic tumor burden and sPD-L1 levels, as well as a significant increase of sPD-L1 during treatment with ICI were demonstrated. PD-L1 can be used as a new biomarker in the early assessment and monitoring of immunotherapy efficacy.

CT: computed tomography; PD: progressive disease; CR: complete response; NSCLC: non-small-cell lung cancer; ICPIs: immune checkpoint inhibitors; iMPERCIST: immunotherapy-modified PERCIST; IrRC: immune-related response; OS: Overall Survival; PFS: progression free survival; iRECIST: immune-related Response Evaluation Criteria in Solid Tumors.
